# Efficacy and safety of atrial fibrillation ablation in patients with Marfan syndrome: A multicenter analysis

**DOI:** 10.1016/j.hroo.2026.03.022

**Published:** 2026-03-27

**Authors:** Hussein Abdul Nabi, Luke Dreher, Hend Bcharah, Christopher Kanaan, Ramzi Ibrahim, Charbel Hatem, Komandoor Srivathsan, Fadi E. Shamoun, Hicham El Masry

**Affiliations:** 1 Department of Cardiovascular Medicine, Mayo Clinic, Phoenix, Arizona; 2 Department of Clinical Genomics, Mayo Clinic, Phoenix, Arizona

**Keywords:** Marfan syndrome, Atrial fibrillation, Ablation, Recurrent atrial fibrillation, Complications, Pacemaker

## Abstract

**Background:**

Patients with Marfan syndrome (MFS) are at an increased risk of atrial fibrillation (AF) and atrial flutter (AFL), yet data on catheter ablation outcomes in this population remain limited.

**Objective:**

This study aimed to evaluate recurrence rates, clinical outcomes, and procedural complications after AF and AFL ablation in patients with MFS.

**Methods:**

We conducted a retrospective study of patients with confirmed MFS treated across Mayo Clinic sites from 2018 to 2024. Among 1113 screened patients, 979 had confirmed MFS. After excluding those younger than 18 years and with insufficient follow-up, 209 patients with documented AF or AFL remained, of whom 67 underwent catheter ablation. Collected data included demographics, arrhythmia characteristics, ablation type, recurrence, complications, and echocardiographic findings.

**Results:**

Among 67 ablation patients, 41 (61.2%) underwent rhythm-targeted AF ablation, 15 (22.4%) cavotricuspid isthmus ablation, and 11 (16.4%) atrioventricular node ablation. Within the AF ablation group, 26 (63.4%) had paroxysmal AF and 15 (36.6%) persistent AF; 85% also had AFL. Recurrence occurred in 76.9% of patients with paroxysmal AF (median 1.03 years) and in 93.3% of persistent AF (median 0.82 years). Repeat ablation was required in 42.3% and 60%, respectively. Overall, 34 of 41 (82.9%) experienced recurrence, and 20 (48.8%) underwent multiple procedures. Complications occurred in 19 (28.4%), including vascular injury, bleeding, retroperitoneal hemorrhage, pericardial effusion, pulmonary vein stenosis, phrenic nerve injury, and thromboembolic events.

**Conclusion:**

In patients with MFS, AF ablation is associated with high recurrence rates, frequent repeat procedures, and notable complication risk. These findings emphasize careful patient selection and vigilant long-term follow-up.

**Key Findings**

▪ Atrial fibrillation (AF) is common in patients with Marfan syndrome (MFS). Among 979 patients with confirmed MFS, 221 (22.6%) had AF or atrial flutter (AFL), highlighting a substantial burden of atrial arrhythmias in this population.
▪ Catheter ablation demonstrated limited long-term efficacy. Among patients undergoing rhythm-targeted ablation for AF, 81% experienced recurrent atrial arrhythmias, with a median time to recurrence of 0.88 years, and nearly half (48.8%) required repeat ablation procedures.
▪ Recurrence rates were particularly high in persistent AF. In patients with persistent AF, 93.3% experienced recurrence after ablation, and 60% required at least 1 repeat ablation procedure.
▪ Procedural complications were relatively frequent. Among patients undergoing ablation, 23.9% experienced complications, including bleeding events, pericardial complications, pulmonary vein stenosis, and thromboembolic events.
▪ Pacemaker implantation and device-related complications were notable. Pacemakers were implanted in 6.2% of patients with AF/AFL, with device-related complications occurring in 23.1% of those receiving pacemakers.

## Introduction

Marfan syndrome (MFS) is an autosomal dominant connective tissue disorder that affects approximately 1 in 5000 to 1 in 10,000 individuals.^1^^,^^2^ In up to 90% of cases, it is caused by pathogenic or likely pathogenic variants in the *FBN1* gene.^1^ MFS is associated with involvement of multiple organ systems, which can include ocular, dermatologic, musculoskeletal, cardiovascular, pulmonary, and central nervous system manifestations.^3^

Physical features of MFS can vary widely and may include dolichostenomelia, arachnodactyly, thoracolumbar scoliosis, pectus deformities, bone overgrowth, joint laxity, lens dislocation, cataracts, myopia, and retinal detachment.^3^^,^^4^ Vascular involvement and complications pose the greatest threat to patients with MFS, particularly the development of aortic aneurysms and dissections, as well as involvement of other vascular territories.^5^ Cardiovascular manifestations also contribute significantly to morbidity and mortality in these patients, including aortic rupture, valvular abnormalities such as aortic regurgitation and mitral valve prolapse (MVP), and tricuspid regurgitation.^4^

With the implementation of appropriate management strategies, regular cardiac monitoring, and timely elective surgical interventions, life expectancy in patients with MFS has increased by 3–5 decades.^5^ However, the clinical significance, efficacy, and safety of electrophysiological interventions in MFS have not been well studied or explored. Previous studies reported a high prevalence of atrial arrhythmias in patients with MFS. For example, in a retrospective analysis of 213 patients with MFS and confirmed *FBN1* variants, atrial arrhythmias were observed in 35 patients (16%), of whom 33 (94%) had atrial fibrillation (AF).^6^ Most patients received beta-blocker therapy, with a substantial proportion requiring antiarrhythmic treatment or ablation procedures.^6^

Although a previous case series involving 4 patients with MFS reported outcomes of catheter-based ablation, showing that 3 patients maintained sinus rhythm after multiple procedures with minimal periprocedural complications, larger cohort studies with detailed evaluation of ablation sites, recurrence rates, and complication profiles are lacking.^7^ This study aimed to assess the effectiveness and safety of AF ablation in this patient population, providing a comprehensive analysis of procedural details and clinical outcomes.

## Methods

### Study population

This retrospective study included patients diagnosed as having MFS between 2018 and 2024 at Mayo Clinic sites in Rochester, Arizona, and Florida. Patients were initially identified using the International Classification of Diseases, 10th Revision, codes, and diagnoses were subsequently confirmed through manual chart review. Individuals younger than 18 years and those with limited follow-up were excluded. This study was approved by the institutional review board, and only patients who provided an informed consent were included. The research reported in this paper adhered to the ethical principles outlined in the Declaration of Helsinki.

### Data collection

Comprehensive chart reviews were conducted to identify patients with a history of AF or atrial flutter (AFL) who had undergone catheter ablation. Initially, 1113 patients were identified using International Classification of Diseases coding. After a detailed chart review, which included genetic testing or phenotypic confirmation, 979 patients were confirmed to have MFS. Among these, 221 patients were found to have a documented history of AF or AFL. After excluding patients with inadequate follow-up, 209 patients remained eligible. Of these, 67 had undergone catheter ablation for AF or AFL and were included in the final analysis.

For these 67 patients, detailed chart review was performed to collect baseline demographic and clinical characteristics, date of AF or AFL diagnosis, ablation procedure dates, total number of ablations, recurrence of AF (including timing), and ablation-related complications. The use of antiarrhythmic medications before and after the first ablation was documented. For patients who underwent atrioventricular (AV) node ablation as the initial strategy, the clinical rationale was recorded.

Echocardiographic data were reviewed for all patients, including both the first and most recent studies. Left atrial volume index and left ventricular ejection fraction (EF) were extracted. EF was assessed using 3-dimensional imaging when available; if not, 2-dimensional measurements and the biplane method were used as alternatives.^8^ Owing to the established association between MVP and connective tissue disorders, all patients were assessed for the presence of MVP.

Chart reviews also identified patients who received pacemaker implantation, either in the context of an AV node ablation strategy or for tachycardia-bradycardia syndrome. Any associated pacemaker-related complications were recorded.

### Study outcomes

The primary objective of this study was to assess the efficacy of catheter ablation for AF in patients with MFS. Secondary objectives included evaluating the safety of ablation procedures, identifying the rate of ablation-related complications, and assessing outcomes related to pacemaker implantation either after ablation or owing to bradyarrhythmia associated with AF.

### Statistical analysis

Statistical analyses were performed using IBM SPSS Statistics (IBM Corp, Armonk, NY). Categorical variables were summarized using frequencies and percentages. Continuous variables were assessed for normality using the Shapiro-Wilk test and presented as mean ± standard deviation for normally distributed data or median with interquartile range (IQR) for non-normally distributed data.

## Results

### Baseline characteristics

**Figure 1:**
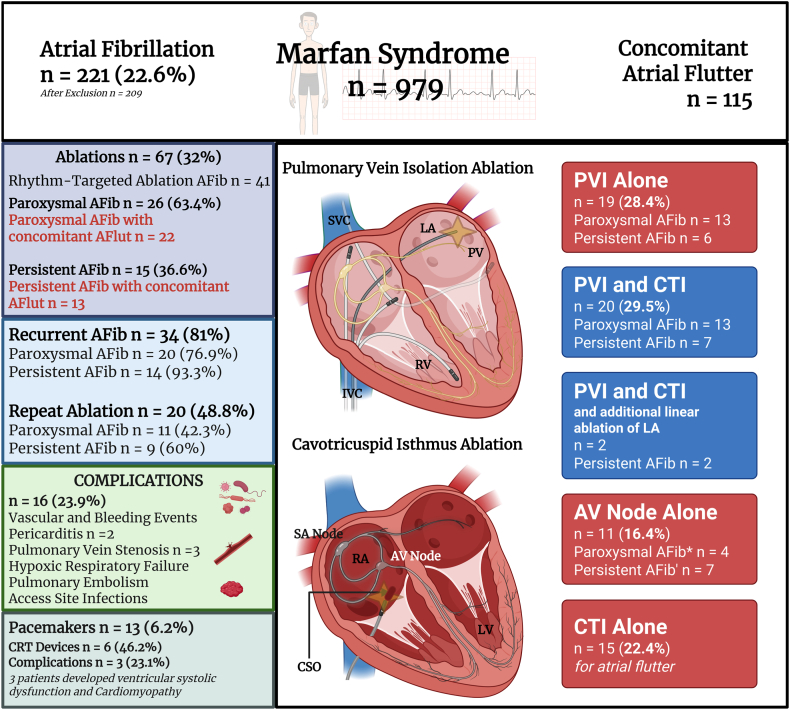
AFib ablation in patients with Marfan syndrome. Among 979 patients with Marfan syndrome, 221 (22.6%) had AFib and 115 had concomitant AFlut. After exclusion, 67 patients underwent 67 ablation procedures, including 41 rhythm-targeted ablations for AFib. PVI-based strategies were the most commonly used approaches, followed by CTI-only and AV node ablation strategies. Recurrence after rhythm-targeted AFib ablation occurred in 34 patients (81%), and repeat ablation was performed in 20 (48.8%). Procedure-related complications occurred in 16 patients (23.9%), and 13 patients (6.2%) required pacemaker implantation, including 6 who received CRT devices. 3 patients developed ventricular systolic dysfunction and cardiomyopathy. ∗Of the patients with paroxysmal AFib who underwent AV node ablation alone (n = 4), 2 of them also had AFlut. ‘Of the patients with persistent AFib who underwent AV node ablation alone (n = 7), 4 of them also had AFlut. “Created in BioRender. Dreher, L. (2026) https://BioRender.com/3yvsww4.” AFib = atrial fibrillation; AFlut = atrial flutter; AV = atrioventricular; CRT = cardiac resynchronization therapy; CSO = coronary sinus ostium; CTI = cavotricuspid isthmus; IVC = inferior vena cava; LA = left atrium; LV = left ventricle; PV = pulmonary vein; PVI = pulmonary vein isolation; RA = right atrium; RV = right ventricle; SVC = superior vena cava.

Among 979 patients identified with MFS, 221 (22.6%) were found to have AF. After applying the exclusion criteria, 209 patients were eligible for screening for ablation procedures. These 209 patients had a median age of 63 ± 18 years (IQR) and were predominantly of white race (187 patients [92.6%]). The cohort included 90 females (43.1%) and 119 males (56.9%). The median left atrial volume index was 39 ± 25 mL/m^2^ (IQR), and the median EF was 58% ± 13% (IQR). The basic characteristics of this population are presented in Table 1. Of these, 67 (32%) underwent at least 1 ablation procedure for atrial arrhythmias, including AF and/or AFL. Among the 209 patients, 115 had concomitant AFL. Within the ablation group, 11 patients (16.4%) underwent AV node ablation, 15 (22.4%) underwent cavotricuspid isthmus (CTI) ablation alone for AFL, and the remaining 41 (61.2%) underwent other types of ablations targeting AF alone or in combination with AFL. An overview of the key findings is presented in Figure 1.

**Table 1:** Baseline characteristics

Variable	Median ± IQR	
Continuous variables		
Age (y)	63 ± 18	
Ejection fraction (%)	58 ± 13	
Left atrial volume index	39 ± 25	
	Frequency (n)	Percent (%)
Categorical variables		
Race (white)	187	92.60
Gender		
Male	119	56.9
Female	90	43.1
Mitral valve prolapse	123	58.9
Hypertension	133	63.6
Diabetes mellitus	24	11.5
Chronic kidney disease	53	25.4
Stroke/transient ischemic attack	43	20.1

IQR = interquartile range.

### Paroxysmal AF ± AFL ablation

Among the 41 patients who underwent rhythm-targeted ablation for AF, 26 (63.4%) were diagnosed as having paroxysmal AF, and of these, 22 had concomitant AFL. Within this subgroup, 13 patients (50%) underwent a combined pulmonary vein isolation (PVI) and CTI ablation. The remaining 13 patients (50%) received PVI alone, with some also undergoing additional linear ablations targeting specific regions of the left atrium, such as the left atrial roof line or an anterior mitral isthmus line. Among patients with paroxysmal AF who underwent at least 1 ablation, 20 (76.9%) experienced AF recurrence, with a median time to recurrence of 1.03 years (IQR 2.16 years). Recurrence occurred within 1 year in 9 patients (34.6%) and within 3 years in 16 patients (61.5%). In addition, 11 patients (42.3%) required 2 or more ablation procedures, with 5 of them undergoing a second ablation within 1 year.

### Persistent AF ± AFL ablation

Among the 41 patients who underwent rhythm-targeted ablation for AF, 15 patients (36.6%) had persistent AF, and of these, 13 had concomitant AFL.

Of the 15 patients with persistent AF, 7 patients (46.7%) underwent combined PVI and CTI ablation, 6 patients (40%) received PVI alone, and 2 patients (13.3%) underwent extensive ablation, including PVI, CTI, and additional linear ablation of the left atrium (eg, roof line and mitral isthmus line).

Among the 15 patients with persistent AF, 14 (93.3%) experienced AF recurrence after at least 1 ablation procedure with a median time to recurrence of 0.82 years (IQR 1.16 years), and only 1 patient remained free of recurrence. Recurrence occurred within 1 year in 10 patients (66.7%) and within 3 years in 13 patients (86.7%). In addition, 9 patients (60%) required at least 1 repeat ablation after the initial procedure, and 3 of them underwent the repeat ablation within the first year.

### Overall summary

Among all 41 patients who underwent rhythm-targeted ablation for AF, 34 patients (81%) experienced recurrent atrial arrhythmias with a median time to recurrence of 0.88 years (IQR 1.19 years). Recurrence occurred within 1 year in 19 patients (46.3%) and within 3 years in 29 patients (70.7%). Overall, 20 (48.8%) required at least 1 repeat ablation procedure. Among the 15 patients who underwent CTI ablation for AFL, 4 patients (26.7%) experienced recurrent typical AFL.

### AV node ablation

AV node ablation was performed as the initial strategy in 11 patients (5.2% of the total cohort; 16.4% of the ablation cohort). This included 2 patients with paroxysmal AF alone, 2 with paroxysmal AF and AFL, 3 with persistent AF alone, and 4 with persistent AF and AFL. Indications included permanent AF with poor rate control, advanced age with multiple comorbidities precluding rhythm control, severely reduced left ventricular EF requiring biventricular implantable cardioverter-defibrillator implantation, and the presence of a preexisting pacemaker. These patients were excluded from rhythm-specific recurrence and redo analyses.

### Procedural complications

Among the 67 patients who underwent ablation, 16 (23.9%) experienced procedural complications. Vascular and bleeding events were most common. These included 1 case of retroperitoneal hemorrhage with hemorrhagic shock requiring vasopressor support with phenylephrine and blood transfusion. The patient was managed conservatively with hemodynamic stabilization and close monitoring without invasive intervention, and hospitalization was prolonged for observation and recovery. Additional vascular complications included clinically significant access-site hematomas. 1 patient developed a large hematoma requiring CT imaging to evaluate for vascular injury; a pseudoaneurysm was identified and managed conservatively with supportive care and observation, resulting in an extended hospital stay for monitoring. Another patient developed bleeding from the right femoral access site with an associated hematoma that was managed conservatively with observation. Anticoagulation-related bleeding attributed to heparin was also observed. Overall, these vascular complications required blood transfusion and resulted in prolonged hospitalization for stabilization and monitoring; however, all patients were successfully managed with supportive care without the need for invasive vascular intervention.

Inflammatory and cardiac complications included 2 cases of pericarditis that progressed to effusion requiring pericardiocentesis and 1 case of left atrial roof perforation, which was managed conservatively but required pericardiocentesis and prolonged hospitalization for monitoring and supportive care, with subsequent clinical stabilization. Pulmonary complications included 3 cases of pulmonary vein stenosis or chronic occlusion and 1 case of pleural effusion requiring thoracentesis. 1 patient experienced phrenic nerve injury resulting in hypoxic respiratory failure requiring BiPAP.

A thromboembolic event was observed in 1 patient who developed an intracardiac thrombus with subsequent bilateral pulmonary embolism. 2 patients developed access-site infections.

### Complications after pacemaker implantation

Pacemakers were implanted in 13 patients (6.2%) (of 209 patients), either before AV node ablation or for AF with a slow ventricular rate. Pacemakers implanted for other indications were excluded from this analysis. Of these 13 patients, 6 (46.2%) received cardiac resynchronization therapy (CRT) devices. Pacemaker-related complications occurred in 23.1% of this subgroup (3 patients); all events occurred beyond the first month. 2 cases were lead related (both owing to lead dislodgement), and 1 patient developed a lead infection. An additional 3 (23.1%) developed ventricular systolic dysfunction and cardiomyopathy attributed to pacemaker-induced desynchrony.

## Discussion

This multicenter study represents the largest cohort to date evaluating the efficacy and safety of catheter ablation for AF and AFL in patients with MFS. Our findings reveal several clinically important observations, most notably the exceptionally high recurrence rate of atrial arrhythmias (81%) and the substantial need for repeat procedures (48.8%). In comparison, recurrence rates in the general population range from 25% to 40%, with repeat ablation rates of 20%–30%, underscoring nearly a 3-fold higher recurrence burden in MFS.^8^, ^9^, ^10^ Procedural complications were also markedly higher in this population (23.9% vs 4%–10% in the general population^10^^,^^11^), including serious vascular, cardiac, and thromboembolic events. Device-related complications after pacemaker implantation were frequent (23.1%), further emphasizing the complexity of rhythm management in MFS.

Literature on ablation in MFS has been confined to small case series and individual reports, which often suggested favorable outcomes with minimal complications. Previous small reports have described encouraging procedural outcomes in MFS, including sustained sinus rhythm after AF ablation in most patients from a 4-patient series, although repeat procedures were often needed, as well as successful CTI ablation and feasible laser balloon PVI.^7^^,^^9^^,^^10^ In contrast, our larger, longitudinal study demonstrates substantially higher recurrence and complication rates. This discrepancy is likely attributable to broader patient inclusion (including older, higher-risk patients) and more complete follow-up, whereas previous reports typically involved younger, highly selected individuals.

Multiple factors intrinsic to MFS may explain these findings. Structural atrial remodeling, particularly in the context of MVP, was common in our cohort (58.9%). MVP produces chronic left atrial volume overload, resulting in enlargement and progressive remodeling, thereby increasing arrhythmogenic substrate and reducing the durability of ablation.^12^ The high prevalence of concomitant AFL (85%) further suggests diffuse atrial disease with biatrial involvement, limiting the efficacy of PVI alone.^13^^,^^14^

Connective tissue fragility inherent to FBN1 mutations may also contribute. Abnormal extracellular matrix composition and impaired wound healing could compromise lesion durability, increasing the likelihood of reconnection and recurrence.^15^^,^^16^ Although direct evidence regarding ablation lesion healing in MFS is limited, these pathophysiological mechanisms are consistent with broader studies of connective tissue biology. In addition, autonomic dysregulation, recently shown to be more prevalent in MFS,^17^ may lower the threshold for arrhythmia recurrence after ablation.

Complication rates were also elevated. Approximately one-quarter of patients experienced ablation-related complications, ranging from vascular injuries to pericardial effusion and thromboembolism. These risks are likely amplified by connective tissue fragility.^18^ Pacemaker implantation also carried unique challenges. Of the 209 patients with atrial arrhythmias, 13 (6.2%) required permanent pacing, with device-related complications including lead malfunctions, infections, and pacing-induced cardiomyopathy. The skeletal deformities common in MFS (eg, scoliosis, pectus deformities) may complicate implantation and contribute to long-term device-related morbidity.^19^

These findings have important clinical implications. Rhythm management in MFS requires meticulous patient selection, shared decision making, and careful assessment of risk vs benefit. Given the high rates of recurrence, repeat ablation, and procedural complications, clinicians should consider conservative thresholds for intervention and incorporate detailed preprocedural imaging (computed tomography or magnetic resonance imaging) to guide decision making. Alternative approaches such as AV node ablation with CRT should be considered early, particularly in patients at risk of ventricular dysfunction.^20^ Although current guidelines endorse AV node ablation with CRT for patients with AF at risk of pacing-induced cardiomyopathy,^21^^,^^22^ no specific recommendations exist for MFS. Likewise, surgical ablation (maze procedure) during concomitant valve or aortic surgery may offer improved long-term rhythm outcomes, survival benefit, and reduced thromboembolic risk.^20^^,^^21^ Further studies are needed to define the optimal rhythm management strategies for this high-risk population.

### Limitations

This study has several limitations. First, as a retrospective analysis conducted across 3 tertiary referral centers, practice patterns may not reflect those in other settings, limiting generalizability. Second, retrospective design inherently introduces selection bias. Third, in some cases, ablation sites were identified from physician notes rather than procedure reports, potentially leading to underreporting. However, this was mitigated by careful cross-verification of electrophysiologists’ documentation.

## Conclusions

Atrial arrhythmias are common in patients with MFS, and catheter ablation in this population is associated with high recurrence rates and an elevated risk of procedural complications compared with the general AF population. Device-related morbidity, particularly with pacemaker implantation, further complicates long-term rhythm management. These findings highlight the importance of individualized treatment strategies, cautious procedural planning, and vigilant follow-up. A personalized, multidisciplinary approach remains essential to balancing efficacy and safety in the management of atrial arrhythmias in patients with MFS.
